# Lived experience of being a grandparent in one region of Spain: a qualitative study

**DOI:** 10.3389/fpubh.2024.1419207

**Published:** 2024-11-27

**Authors:** Sofia García-Sanjuán, Ana Isabel Gutiérrez-García, María José Cabañero-Martínez, Manuel Fernández-Alcántara, Maria del Carmen Rocamora-Rodriguez, Silvia Escribano

**Affiliations:** ^1^Department of Nursing, Faculty of Health Sciences, University of Alicante, Alicante, Spain; ^2^Institute of Health and Biomedical Research of Alicante (ISABIAL), Group of Person-Centred Care and Innovation in Health Outcomes, University of Alicante, Alicante, Spain; ^3^EYCC Research Group, University of Alicante, Alicante, Spain; ^4^Department of Health Psychology, Faculty of Health Sciences, University of Alicante, Alicante, Spain

**Keywords:** caregiving, grandchildren, grandparents, life experience, qualitative research

## Abstract

**Background:**

The evolution of the family model in Europe in the 21st century, and particularly in Spain, has led to grandparents playing a major role in caring for their grandchildren. Grandparents are required to take on certain functions and roles in order to provide this care. This results in changes to their daily lives, their family relationships and therefore their quality of life.

**Objective:**

To explore grandparents’ lived experience of being involved in the upbringing and care of grandchildren and to determine how this affects their quality of life.

**Methodology:**

A descriptive qualitative design was used. The data collection strategy involved two focus groups in two health centres in the province of Alicante (Spain), which were attended by 19 grandparents. Thematic analysis was used to analyse the data.

**Results:**

Four themes and their respective sub-themes emerged from the data analysis: (1) Not only caring, but also raising (implications for upbringing; dealing with their children’s rules; nutrition of grandchildren); (2) Motivation for providing care (it is what families do; financial support; barriers to caregiving that outweigh the reasons for caregiving); (3) Significance of gender (grandmothers bring up, grandfathers help out; cultural burden of caregiving for women) and (4) Implications of care (negative aspects of caregiving; positive aspects of caregiving).

**Conclusion:**

The study findings show that while grandparents recognise the value and benefits of providing regular childcare, there are important challenges that need to be addressed. It would therefore be advisable for health professionals to take into account the experience of grandparent caregivers and the process of caring for young children when developing inclusive policies for this population of caregivers.

## 1 Introduction

The family is a global institution of a historical and dynamic nature. It consists of a series of interconnected networks that contribute to the development of the individual and the acquisition of prevailing social values. However, throughout history, the family has undergone various transformations, both in its structure and in its relationships. The pace and intensity of these changes have varied from one society to another and have influenced the way its members relate to each other (1, 2).

Most Spanish families in the 20th century adhered to a patriarchal structure and the notion of permanence of marriage, closely linked to the Catholic faith, in which the care of the youngest members of the family fell to the figure of the mother, with no need for child-rearing assistance (3). However, this traditional model has rapidly evolved towards a contemporary model of family, especially affected by the incorporation of women into working life, causing the model to undergo a change and increasing the active participation of grandparents in the upbringing and care of their grandchildren (4).

The reasons for this change in the family model, with grandparents as the core caregivers of the youngest children, are many and complex, and are intertwined with various socio-demographic indicators such as the ageing of the population (5). This demographic trend is leading to a greater concentration of older adults who are in better health and therefore able to provide ongoing care for their grandchildren. In this way, their participation in their role in the family goes beyond caregiving and they take on parenting (6). This phenomenon, whether occupying a central or peripheral role, is known as “grandparenting” (4) and is characterised as a relational dynamic that links two non-adjacent generations - grandparents and grandchildren - mediated by another generation of parents who happen to be their own children, i.e., their direct filiation (7).

Compared to other European countries, Spain is one of the countries with households built on strong family ties and with a more deeply rooted familistic culture (8). Here, intergenerational relationships continue to play a crucial role as a pillar of social wellbeing and therefore rely on the support of grandparents as caregivers for their grandchildren, partly because parents work long hours and do not have adequate time for child-rearing. The Survey on Health, Ageing and Retirement in Europe (SHARE) (9) shows that 22.7% of grandparents care for their grandchildren. Moreover, the intensity and circumstances of grandparental care vary widely and are influenced by a range of family dynamics (10, 11).

Caring for grandchildren has a significant impact on the grandparents’ health. This impact will be regulated by different factors, including the intensity of care, i.e., the number of hours spent caring for grandchildren. So-called “custodial care,” where grandparents take responsibility for their grandchildren for more than 12 h a day, appears to have a negative impact on grandparents’ health (12–14). In contrast, supplementary or occasional care, typically between 15 and 20 h per week, appears to be beneficial to grandparents’ health. Benefits include maintaining greater physical mobility, improved life satisfaction and lower levels of depressive symptoms (15, 16).

Another factor to consider that affects the health of grandparents caring for their grandchildren is related to work and economic aspects of the grandparents’ lives. Grandparents with fewer financial resources or who have reduced their working hours to care for their grandchildren are more prone to depression (13). It should also be noted that grandparents who take care of their grandchildren often contribute directly or indirectly to their children’s household finances, thus depleting their own financial resources (6).

Other variables, such as the age of grandparents and grandchildren, also influence the health implications of caregiving. Younger grandparents feel more useful when providing care, but the older the grandparent, the greater the risk of adverse health effects (17) intergenerational dynamics within each family system must also be taken into account. Grandparents, whose roles are often undefined or poorly understood, may feel overwhelmed or overburdened by this (18).

The efforts of grandparents in Spain often go unnoticed. This is because there is still a strong intergenerational bond between grandparents and grandchildren and, in general, the grandparent-grandchild relationship seems to have a positive impact on grandparents (4). Most of the research that has been conducted in Spain has focused on examining specific variables such as physical, economic and/or emotional factors and their impact on grandparents‘health at a given time in a quantitative way, without delving into the grandparents’ experiences of providing this upbringing. On the other hand, some studies have analysed the effects of caring for grandchildren on the formation of intergenerational relationships and the specific factors that predispose older people to take on the role of caregiver for their grandchildren. However, these studies have tended to overlook the lived experiences of grandparents in assuming these responsibilities (4, 19). To our knowledge, there are no qualitative studies documenting the experiences of Spanish grandparents caring for their grandchildren. Therefore, the aim of this study was to explore grandparents’ lived experiences of being involved in the upbringing and care of their grandchildren, and to determine how these practices affect their quality of life.

## 2 Methodology

### 2.1 Design

We adopted a descriptive qualitative approach using focus groups as a data collection strategy to better understand the impact of caring for grandchildren on quality of life (20). This method was chosen because focus groups are a particularly fruitful way of stimulating discussion and collecting rich data on a particular set of issues. Focus groups also tend to encourage the participation of people who would not normally take part in certain discussions (21).

### 2.2 Participants

The study population included caregivers of grandchildren in the Torrevieja area [Guardamar Health Centre (HC)] and the Elda Health Department (Acacias HC), Alicante (Spain). The inclusion criteria were: people attending routine visits to the nurse in the district’s health centres, who were grandparents, who spoke and understood Spanish, who were not frail or had any functional or sensory impairment, and who were caring for grandchildren without any degree of dependency or incapacity. Older adults with cognitive or other impairments that made communication difficult were excluded, and two people who were cohabiting or married and thus sharing care were not allowed to contribute to the same focus group. A total of 19 participants took part in the study. The majority were women and the mean age was 67.15 years (Table 1).

**Table 1:** Socio-demographic data from the focus groups.

	Age	Gender	Marital status	Employment status	No. of children	No. of grandchildren	Hours of care	Years of care
PG1	67	M	Married	Retired	3	5	45	3
PG2	69	M	Widowed	Retired	2	1	15	3
PG3	63	F	Married	Retired	2	3	30	11
PG4	66	M	Married	Retired	2	2	25	6
PG5	66	M	Married	Retired	2	3	6	6
PG6	69	F	Married	Retired	3	3	30	10
PG7	66	F	Married	Retired	2	3	10	6
PG8	66	F	Married	Working full time	3	4	40	4
PG9	74	M	Married	Retired	2	4	35	10
PG10	69	F	Married	Working full time	2	3	40	14
PE1	68	M	Married	Retired	2	5	15	11
PE2	68	F	Married	Retired	4	6	10	7
PE3	67	F	Married	Retired	2	2	30	14
PE4	72	F	Widowed	Retired	3	7	20	14
PE5	63	M	Married	Unemployed	2	2	10	7
PE6	58	F	Married	Working full time	2	2	15	4
PE7	67	F	Married	Retired	2	2	20	7
PE8	69	F	Married	Working full time	3	2	25	10
PE9	69	M	Married	Retired	2	3	30	12

“PG” Guardamar Health Centre Group; “PE” Elda Health Department Group.

### 2.3 Instruments

**Figure 1:**
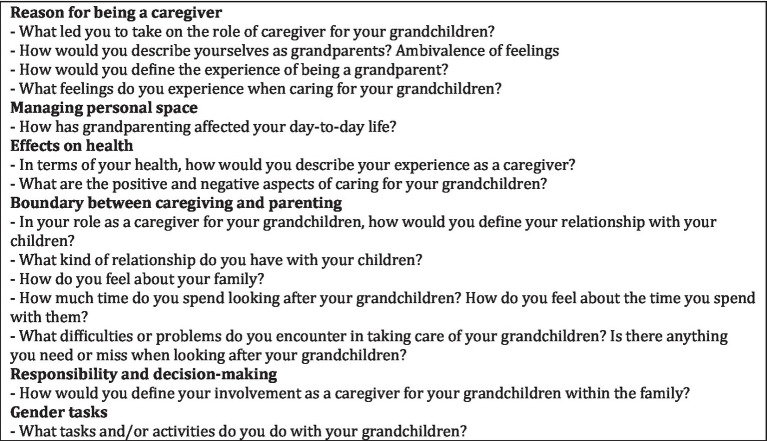
Focus group guide script.

We prepared a questionnaire to collect the participants’ socio-demographic information. We also developed a script of open-ended questions to facilitate and guide the focus group. The questions were drawn from the existing literature on the research topic (Figure 1).

### 2.4 Procedure

Healthcare staff at each health centre provided the lead researcher with the contact details of people who fulfilled the inclusion criteria. Having obtained the information about potential participants, the researchers telephoned them to give them more information about the study and explain what their participation would involve, with a view to arranging a face-to-face appointment for the focus group. The researchers contacted 30 potential participants from the two centres. However, 11 of them declined the invitation, either due to time constraints or health problems. We eventually recruited 10 participants for the Guardamar HC Group and nine participants for the Elda HC Group.

We held two focus groups between October and November 2021 in specially prepared rooms at the two health centres. The first session (Guardamar HC Group) was approximately 105 min in duration, while the second session (Elda HC Group) was approximately 95 min. Two researchers trained in qualitative research, with no prior relationship to the participants, moderated the groups. One took the role of facilitator and asked questions of the group, while the other made field notes. The groups discussed each topic separately until a consensus was reached. With the consent of the participants, the focus group discussions were videotaped for later analysis.

### 2.5 Data analysis

Both the systematic processes used to obtain data and the subsequent data collection contribute to the validity of qualitative research (22). In conducting the focus groups, the lead interviewer (GS) ensured that the resulting data were consistent and on topic. Data analysis was guided by Braun and Clarke’s method of Thematic Analysis (23). SG and AG analysed the focus group transcripts using an open and inductive coding system in which each paragraph or sentence was given an emergent code summarising its meaning. Cases of disagreement were settled by a third party (MJC). The codes were organised into groups based on their similarity to each other. Having identified these patterns in the interviews, we then grouped them into themes and sub-themes. After reflecting on the differences and comparisons with the available academic literature on the subject, as well as the conceptual framework of this paper, we refined the different themes and sub-themes and determined which data could best be used to make sense of them (23). Verbatim quotes (in italics) have been included in the manuscript of this study to illustrate the different themes. Each quote is identified by the code assigned to each participant in both focus groups.

## 3 Results

From the data collected on the experience of caring for grandchildren, we have identified four themes and their corresponding sub-themes: (1) Not only caring, but also raising (the grandchildren); (2) Motivation for providing care; (3) Significance of gender; (4) Implications of caregiving (Table 2).

**Table 2:** Themes and sub-themes.

Themes	Sub-themes
1. Not only caring, but also raising (the grandchildren)	1.1 Implications for upbringing 1.2 Dealing with their children’s rules 1.3 Nutrition of grandchildren
2. Motivation for caregiving	2.1 It is what families do 2.2 Financial support 2.3 Barriers to caregiving that outweigh the reasons for caregiving
3. Significance of gender	3.1 Grandmothers bring up, grandfathers help out 3.2 Cultural burden of caregiving for women
4. Implications of caregiving	4.1 Negative aspects of caregiving 4.2 Positive aspects of caregiving

### 3.1 Theme 1: Not only caring, but also raising

#### 3.1.1 Implications for upbringing

The discussion began with the issue of raising grandchildren, not just providing care. It then moved on to specifically consider the health implications of caring for grandchildren. On the issue of caregiving, participants agreed that it is not just about spending time with grandchildren, but also about bringing them up. Our interviewees explained that caring for their grandchildren was not limited to picking them up from school or taking them to extra-curricular activities, but that there were aspects of caregiving that went beyond simply ferrying them from one place to another.

PE1: *‘When you have them around for so long, there comes a point where you are playing the role of parent, or you need to be. I mean, my son-in-law is away for months on end and you see him on a video call or he pops in for a few days and then he’s off again…’.*

PG10: *‘And when you realise that you are around them so much and you see things that you would not condone, you step into the parents’ shoes and your children become the grandparents, when they come to pick them up in the evening and you realise that you are being the mum, I tell my kids that I want to be the grandma, not the mum’.*

The participants concluded that they are very involved in their grandchildren’s education, not only by helping them with their homework, but also by teaching them values and basic rules that influence their behaviour. In fact, most of them reported disciplining their grandchildren in the same way as their own children when necessary. The childrearing role of grandparents is therefore a crucial factor in the upbringing and development of their grandchildren.

PG4: *‘How do I raise them? Like I did with my own kids, exactly the same, and when they say something to me, I say this to my grandson, who’s in charge at home? Your grandpa, well, you have to pay attention to what you see here’.*

PE9: *‘Sometimes you have to be firm and other times not so much, and I’m telling you I think I did a good job bringing you up and the same goes for them, and I think they should be allowed to do whatever…’.*

PE7: *‘Yes, and most kids today are being brought up by us, the grandparents, and so we pass on our values and our outlook on life to them’.*

#### 3.1.2 Dealing with their children’s rules

Grandchildren are subject to the rules that govern their grandparents’ lives. Sometimes, however, participants admitted to disagreements with their own children, who try to enforce standards of care that do not always match their own ways of doing things.

PE6: *‘…And I say (to my son), there’s a simple way to fix this, when I have her, I do what I want and you, when you have her, do what you want… I think what I’m doing is following the same rules I followed with you and you know what I’m like, if you do not like it, then do not leave her with me, think about it… we all have to have our own rules’.*

PG3: *‘So she (her daughter) says that you should not hit them, that violence is not the way, that you have to tell children “this no, that yes,” in other words they do not want us smacking them. But hey, if they have it coming, then they need a good smack, but of course, when they (her daughters) were little, there was no violence or anything like that, was there, eh?And look how well they have turned out’.*

PG10: *‘I reckon they have them doing a lot of things after school. Used to be they’d go out and play in the park and stuff, but they are not my kids so I cannot really say anything’.*

#### 3.1.3 Nutrition of grandchildren

The situation is similar when it comes to nutritional arrangements, as most participants have to look after their grandchildren at mealtimes, be it lunch or afternoon snack/dinner. Both groups agreed on the importance of instilling healthy eating habits and also said that they fed their grandchildren according to their own beliefs and norms.

PE7: *‘When I pick them up from school at midday, they have to come home for lunch, so my wife makes lunch for everyone’.*

PG9: *‘Mine have lunch in the school canteen, but of course we give them their afternoon snack and sometimes their dinner, and obviously they eat the same as us’.*

PG1: *‘I even give him breakfast because they bring him to me before they go to work so he can have breakfast and then I take him to school’.*

However, a two-way discussion developed. The first problem was that the children gave advice to their parents about which foods were the best for their children and which were not. Despite these recommendations, grandparents displayed enough judgement to comply or not with their children’s dietary recommendations, depending on their own beliefs. The other aspect of the discussion concerned giving in to the consumption of certain foods. Even though they knew it was not always healthy for their grandchildren, they made concessions simply because they were grandparents and wanted to give their grandchildren a treat. Most were in favour of this and also commented that they would not have taken such liberties with their own children, but were allowed to do so as grandparents. There was also a consensus that grandparents felt that grandchildren ate better with them than with their parents, possibly because they have more time to spend on cooking or grocery shopping. This is in contrast to their children who, due to work commitments, have to rely on quicker and less healthy meals.

PG6: *‘Especially my daughter, who tells me to give them (her grandchildren) lots of fruit and not to give them anything sugary, but come on, the little ones eat the same things we do and if they do not like it, then do not leave them with me’.*

PE2: *‘I do not let them have sweets, but I did not with my own kids either, they used to moan a lot about people bringing sweet things to school, when at home it was all toast and milk and sandwiches… but sometimes I do buy them some sweets’.*

PE8: *‘I think they eat worse at home, when they are with me of course I always make sure they have proper home-cooked meals and at their own house they eat whatever they can get their hands on…’.*

PE3: *‘Yes, absolutely, my grandson will not touch a chickpea unless I make them for him, if I peel the chickpeas and mash them up, then he’ll eat them… they eat worse, one day he said he did not want lentils, I made them, and he ate them…. they are always in a rush and they think that if they do not eat, they’ll have a snack, my son has told me this a thousand times. My granddaughter eats a lot worse when her parents are around’.*

### 3.2 Theme 2: Motivation for caregiving

#### 3.2.1 It is what families do

Group participants quickly came to a consensus that caring for their grandchildren was a matter of tradition, just as they themselves had been cared for and raised by their grandparents. However, there was a difference of opinion in both groups when it came to deciding whether this tradition of family caregiving could be seen as an obligation. While there was some debate about the notion of obligation, it was agreed that despite having to provide care, the majority did not see this as a negative thing. Rather, they saw it as something that had been a normal part of their upbringing since childhood. They looked after their grandchildren because that is what families do, given their understanding of the concept of family in the broadest sense. For our participants, it is a way of showing love, providing support and helping other members of the wider family.

PG3: *‘We have to help our children because they work and we have to help them, that’s the way it’s always been and that’s the way it should be, families have to help each other… and I do not know, the fact is you do not think about it, you just know you have to do it and that’s all there is to it’.*

PE1: *‘Out of obligation, out of devotion, but out of obligation, I’m speaking for myself… out of devotion too, I’m delighted with her, she gives me energy, as I like to say… but at the same time it’s out of obligation…’.*

PG7: *‘They never asked me if I wanted to look after the kids or not, it just sort of happened naturally. Little by little you see that they need help and you step up, but that’s normal. My daughter does check to see if I can take them on this day or that day, but my son calls and straight out tells me what we have to do, but it’s only natural, because if they are working I’ll take the kids and I do not mind’.*

PG6: *‘Yes, it’s true, my grandmother was also there for me and my sisters, more than my parents, just like now it’s our turn to look after them, so we try to do the best we can, as far as possible, just like they did for us’.*

#### 3.2.2 Financial support

The issue of financial support was also discussed at this point. All participants were in agreement that if they did not take on the role of caregiver, their children would have to pay for external providers and this would have a negative impact on the family’s finances. However, some participants observed that although their children could afford paid childcare, it was they themselves who offered to look after their grandchildren, even when it was not financially necessary. Once again, this underlined the notion of caregiving being a family tradition.

PE2: *‘If I could not do it, they’d have to pay someone else to look after them, so if we can help our children, it’s the best thing we can do, and besides, it’s no hassle for me to look after my grandchildren, even if sometimes it’s really tiring or you do not feel well one day…’.*

PG7: *‘I was the one who made the decision to take my granddaughter. I mean, who were they going to leave her with, a stranger? Well, I was happy to have her and I’m also saving them money because they are strapped for cash, so it’s good for them and it’s good for me too because I love hanging out with her’.*

#### 3.2.3 Barriers to caregiving that outweigh the reasons for caregiving

It was also noted that some of the participants, although willing to look after their grandchildren, found it impossible to do so. These were very old people with various health issues that prevented them from having the mobility to run after their grandchildren, or they were physically exhausted from the daily demands of caring for their own parents. They also reported facing challenges in using new technologies while providing care. There is a clear generation gap between grandparents and grandchildren, and grandparents often find it difficult to maintain control of such devices. This creates a communication gap that acts as a barrier when it comes to taking care of and supervising younger children.

PE8: *‘I have fibromyalgia and there are days when I cannot be with them, I’m stuck on the sofa, but my husband takes care of them and I try to do what I can’.*

PG5: *‘After a hard day’s work, at my age, I’m worn out and guess what, I’ve got to look after them, there are days when I’d rather just crash when I get home, but I’ve got to look after them and that’s all there is to it’.*

PE3: *‘They grab my phone and I do not let them, because then they put stuff on it that I do not know how to get rid of, so if they need a phone for something, they should use their parents’ phones when they are at home’.*

PE6: *‘The trouble is, they just do not know how to play outside, they are glued to their phones all day and get bored if you tell them to do something else, so I give them an old mobile phone of mine to keep them entertained, but I do not really know what they are getting up to either’.*

### 3.3 Theme 3: Significance of gender

#### 3.3.1 Grandmothers bring up, grandfathers help out

Although it was clear from the conversations that it was the women who were responsible for raising and feeding the grandchildren, it was not until the specific question of ‘*What tasks and/or activities do you do with your grandchildren?*’ that this topic was properly explored. The women in the group were responsible for meals, homework and raising their grandchildren, while the men were responsible for dropping them off and picking them up from school, taking them to after-school activities, if necessary, as well as doing the grocery shopping as directed by their female partners. However, when it came to taking the children to the park, they either did this together or it was just the women.

PE1: *‘If they are sick, I take them to the doctor… the truth is that I’m more responsible for taking them to school and wherever they need to go, but my wife is the real caregiver, so I do not feel that responsibility that you are talking about… maybe it’s because you are women and you spoil them more and have to be on top of everything’.*

PG6: *‘Because mine (husband) is oblivious to everything, they come to eat and the three of them get together and there I am in the middle of making lunch and the three of them are sprawled on the floor and so I pick them up and put them on the sofa, but not him, no… because he just shuttles them back and forth and I’m the one left doing all the heavy lifting’.*

PE9: *‘In my house it’s more or less the same, I take them everywhere and my wife looks after meals and snacks, homework and all that, but sometimes you have to put your foot down because they do not want something or cannot be bothered to do something else…’.*

#### 3.3.2 Cultural burden of caregiving for women

Participants soon agreed that women were responsible for the more educational tasks, while men were in charge of the more fun activities. The men acknowledged that this was also a feature of their own parenting and that they continued in this way out of tradition. Moreover, the women also admitted that they were partly to blame for encouraging this type of behaviour because it was what they had been expected to do. It is also worth noting that both genders concluded that their children were more likely to communicate with the women (grandmothers) when it came to giving instructions on how to look after their own children. This further perpetuated the tradition that it is the role of women to look after young children.

PE4: *‘Maybe it’s our own fault, but hey, that’s the way it’s always been with us, so we go along with it, I’m always keeping an eye on them and my husband does the school run and goes shopping for whatever we need, but that’s as far as it goes’.*

PE1: *‘But you are partly to blame for that (being on top of everything the grandchildren do) because you are always hovering over them and never let anyone else do anything, plus my wife cannot say no to my daughters either and is always there for them, no matter how tired she is’.*

### 3.4 Theme 4: Implications of caregiving

#### 3.4.1 Negative aspects of caregiving

All the participants expressed their joy at being grandparents. However, from the very beginning of the discussion on this topic, differences of opinion emerged between the joy of being grandparents and the obligation to take care of their grandchildren. Most participants reported that none of their children had consulted them about the possibility of becoming grandparents, bearing in mind the immediate impact it was to have on their lives. Participants agreed that their children did not ask if they were willing or able to look after their grandchildren, but simply took it for granted. This assumption had negative consequences at times, with some participants observing that caring for their grandchildren meant that they could not devote time to their own lives. This issue proved more controversial than the previous ones, with similar numbers of people in both groups saying that caring for their grandchildren sometimes felt like an unexpected burden. For example, it prevented them from enjoying age-appropriate leisure activities, or meant that they had to push their bodies to do physical activities that they were barely able to cope with by this point.

PE8: *‘It’s like I’m saying, becoming a grandmother for the first time was really exciting for me, but then of course you realise that you have to keep on taking care of them and I’m more tired when I come home from work, but well, they bring them to me in the afternoon and I’m happy to have them, although sometimes I say… Oh, how nice it would be if they did not bring them here this afternoon… I could have a chance to rest’.*

PG10: *‘Even though I do not have much time and I’m tired, I really like being with my grandchildren, if I did not look after them, I might not see them or my children, so it suits me fine, although there are days when I’d rather not have them around and just rest… but hey ho, that’s what we have signed up for now, we’ll do other things when we can’.*

#### 3.4.2 Positive aspects of caregiving

At the same time, there was a group who, despite not having been asked if they were available to look after their grandchildren, insisted that it was a very positive experience for them because it kept them occupied and also forced them to stay physically active, admitting that otherwise they would not leave the house. On a positive note, they also commented that caring for their grandchildren had brought them closer to their children, as their relationship with their children was practically non-existent before they were asked to care for their grandchildren. However, this relationship was sometimes adversely affected when they were unable to agree on the rules and habits that the parents wanted to impose on their children while they were in the care of the grandparents.

PE2: *‘And even if we do not realise it, sometimes I’ve stopped to think and said “if it wasn’t for this, I’d be preoccupied with this, that or the next thing,” this way I do not worry about other stuff, I look at the little one and say “oh look, he’s scratched his face…”. it’s like the brain kicks into overdrive… He falls and hurts his knee, so suddenly you are like, “What happened to him, what did not happen to him?” Instead of watching the football in the afternoon, you are trying to find out what happened to the little guy, it’s like your mind switches gears and you become more active yourself…’;*

PG7: *‘They can be a handful, but they also make you feel energised, and I’m a very happy Grandma to have them come here’.*

PG4: *‘I do not feel like I’ve given up anything important to look after my grandchildren, I feel that taking care of them has given me a lot, more joy, more energy, I used to joke: “they have got me off my medication!” Because they are so much alike, I’ve had some tough summers, everyone to the beach, pool time, back home, shower them, then back to the beach in the afternoon… yes, since they were little, I’ve made them understand that this is your drawer, swimsuits off and into the bathroom!…’.*

After several rounds of discussion, the participants agreed that if the grandparent was in good health, had no desire for leisure activities and did not spend more than 20 h a week caring for the grandchildren, then the impact of caring for them was positive. However, it was also agreed that if caregiving exceeded 15–20 h per week, or if the grandparent had health problems or wanted to engage in age-appropriate activities, then caregiving had a negative impact. Nevertheless, as discussed in the previous sections, they continued to do it anyway because it was the family tradition.

## 4 Discussion

The aim of this study was to explore grandparents’ experiences of being involved in the upbringing and care of their grandchildren and to determine how this affects their health-related quality of life. Our key findings show that there is more to grandparental childcare than merely providing supervision, but that it also involves the transmission of values, education, role-sharing and both a positive and negative impact on their lives.

As in other studies (24, 25), our findings show how grandparents not only look after the young children when the parents are not around, but also have to take responsibility for their upbringing and set standards equivalent to those they set for their own children. In this way, the grandparent figure acts as a role model, providing valuable advice and giving their grandchildren a sense of history and continuity (26).

Taking on this new role affects their relationship with their children as they struggle to reconcile their own parenting models with the parenting styles advocated by their children (27, 28). In effect, they can be said to be exercising their role not as grandparents but as parents, albeit taking into account their children’s recommendations. This means setting clear boundaries with them in a way that does not disrupt family harmony and is compatible with the parenting model of both generations when it comes to looking after grandchildren (16, 29). This role reversal can be a source of stress when caring for grandchildren. Grandparents may disagree with their children’s parenting advice and instead choose to use the same parenting model with their grandchildren that they used with their children (30).

A specific example of this would be nutrition and feeding, a responsibility that often falls to grandparents. While they are aware of and agree on the importance of instilling healthy eating habits, they take issue with their children second-guessing their judgement and dispensing instructions and “orders” about what the grandchildren should eat (31). However, most grandparents agree that their grandchildren eat better with them because they spend more time preparing meals, from grocery shopping to cooking. They often make concessions for their grandchildren that they would not have made for their own children, such as letting them indulge in unhealthy treats. This is also consistent with the literature, which supports the idea that parental feeding practices and rules–and the resulting habits of grandchildren–can sometimes hinder their efforts to promote healthy eating. Similarly, grandchildren may be reluctant to accept new foods because they expect to be served the same foods they eat at home with their parents (32, 33).

Another theme that emerged from our data is the belief that caregiving is seen as a tradition rather than a duty or obligation. In the context of the traditional family, caregiving is regarded as an expression of love, help and support for other members of the wider family (34). This support takes the form of helping their children financially, even if they can afford paid carers. This idea is supported by the existing literature, which suggests that grandparents whose children are less well-off assume even more caregiving hours and responsibilities (25, 35). However, despite these motivations and family traditions, our sample shows that there are limitations and barriers to caregiving when grandparents face age-related physical problems and difficulties using new technologies. These challenges can even affect communication and relationships with their grandchildren (36, 37).

Gender differences are also evident, in line with much of the literature (38). Grandmothers tend to play more of an educational role (meals, homework, childrearing, etc.), while grandfathers are responsible for more practical or recreational activities (ferrying children to and from school and/or after-school activities, going to the park, etc.). These gender differences in the division of childcare responsibilities are a widespread phenomenon internationally, with several aspects having been documented. Firstly, there is a gender gap in terms of the time grandmothers spend caring for their grandchildren compared to grandfathers, even when they live together. Secondly, the gender disparity is reflected in the different tasks assigned to each role (39, 40).

A further theme that emerged from our findings was the perceived consequences of grandparental care. In terms of the negative consequences, we found that the groups in our study referred to the sense of obligation involved in caregiving, with it being less of a voluntary choice and more of an imposition that was taken for granted. This is compounded by the feeling of not being able to focus on their own lives and the impossibility of pursuing leisure activities or other projects (41). However, there are positive aspects related to keeping grandparent carers active and mobile (42–44). It is emphasised that these positive outcomes are more pronounced when grandparents are in good health, when there is no desire to pursue leisure activities, and when the number of hours of childcare is <20 per week. This adds to findings from empirical studies in other countries, which have found positive associations between non-intensive grandparenting and better health outcomes (45–47). However, due to the heterogeneity of the research models used in these studies, it is not possible to state categorically whether caring for grandchildren has positive or negative consequences without taking other factors into account (48).

### 4.1 Limitations

The data analysed in this study reflect the lived experiences of a group of grandparents who provide care for their grandchildren. Looking at this from the perspective of experiential learning within a specific population means that no overall generalisations can be made. The findings presented must be considered in the context of the limitations of this study. Firstly, this study is based on data collected on grandparental childcare at a single point in time, without standardising the length of time spent providing care or the number of grandchildren cared for. It may be of interest for future lines of research to explore the point at which someone becomes a grandparent caregiver for the first time. The sample consisted of 19 people, so a larger sample may be able to provide a more complete insight into caregivers’ experiences. However, our sample represents a range of grandparental childcare experiences at different stages of caring for grandchildren. Therefore, our findings are transferable to other caregivers. Despite its limitations, this study may be useful in helping to understand the perspectives of grandparent caregivers. It provides preliminary evidence that can be used to develop culturally sensitive support programmes or interventions for caregivers in this context.

## 5 Conclusion

Our qualitative study describes the lived experience of grandparents providing care for their grandchildren and explores how this affects their quality of life. It shows how caring for grandchildren obliges them to reassume a parenting role and to oversee the upbringing of their grandchildren in the broadest sense. Grandparents also have to contend with family dynamics and the physical and mental aspects of ageing. However, there is a need for further research into the relationship between grandparental care and grandparents’ health, and the relationship between grandparents’ health and that of their grandchildren, from the perspective of grandparents’ own lived experience. It would therefore be advisable for health professionals to take into account the experience of grandparent caregivers and the process of caring for young children when developing inclusive policies for this population of caregivers.

### 5.1 Implications for practice

The findings of this study have important theoretical and practical implications. From a theoretical standpoint, this study adds to the existing literature by highlighting the central role that grandparent caregivers play within the family structure and shedding light on the complex dynamics involved. In practical terms, our findings underline the need to recognise the social contribution of older people in supplementing childcare and supporting family development. Politicians and public institutions should provide social support to older people who care for their grandchildren, encouraging them to maximise the benefits for the family and to support their own healthy ageing process. It is also important for older caregivers to recognise the contribution they can make to the development of their children’s families. Moreover, cultivating positive attitudes towards ageing is essential for healthy ageing, which may be achieved through their active participation in society.

## Data Availability

The raw data supporting the conclusions of this article will be made available by the authors, without undue reservation.
